# Inferior and Superior Vena Cava Reconstruction

**DOI:** 10.1007/s00270-024-03867-x

**Published:** 2024-09-24

**Authors:** Rick de Graaf, Arne Estler, Gerd Grözinger

**Affiliations:** 1 Department of Diagnostic and Interventional Radiology, Clinic of Friedrichshafen, Friedrichshafen, Germany; 2 Department of Diagnostic and Interventional Neuroradiology, University Hospital Tübingen, Hoppe-Seyler-Str. 3, 72076 Tübingen, Germany; 3 Department for Diagnostic and Interventional Radiology, University Hospital Tübingen, Tübingen, Germany

**Keywords:** Vena cava, Obstruction, Intervention, Venous, Stent, Venoplasty

## Abstract

Obstructions of the superior and inferior vena cava are prevalent etiologies of deep venous obstruction, presenting a spectrum of clinical manifestations ranging from life-threatening conditions to asymptomatic states. The etiological diversity inherent to these central venous obstructions necessitates a subtle approach to their diagnosis and management. This discrepancy in clinical presentations emphasizes the importance of a differentiated diagnostic and therapeutic strategy, tailored to the specific form of vena cava obstruction encountered. This article aims to delineate the various manifestations of vena cava obstruction and encourages specific diagnostic and treatment pathways.

## Introduction

**Fig. 1:**
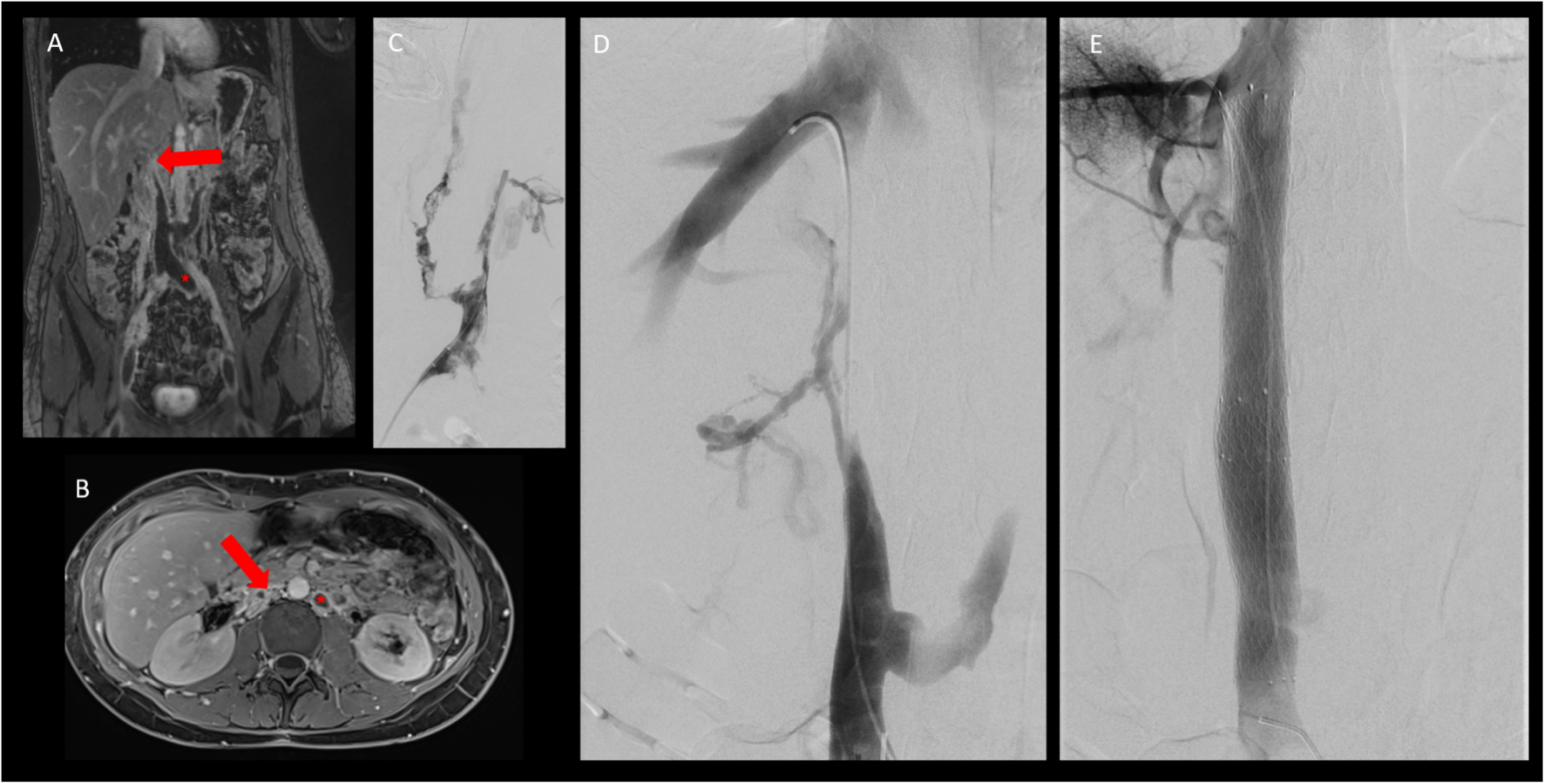
20-year-old female patient with suspected agenesia of the inferior vena cava (VCI) and extensive acute thrombosis of the pelvic veins; **A** and **B**: coronal and axial contrast-enhanced T1 MRV shows extensive acute thrombosis of the pelvic veins (red star in A and B) including thrombosis of venous lakes and lumbar veins; red *arrow:* remnant of IVC, **C**: Phlebography for catheter-directed thrombolysis confirming acute thrombus (patient in prone position). **D**: Chronic occlusion of the supra- and infrarenal part of the IVC after thrombolysis. Passage of the occlusion with catheter in the right hepatic vein. **E**: Final run with reconstructed IVC after Implantation of two 24 mm Sinus XL Stents (Optimed GmbH, Ettlingen, Germany)

SVC obstruction is often referred to as “SVC syndrome” in the literature and affects about 15,000 patients in the USA annually [1]. Malignancy is still the leading cause of SVC obstruction up to 90% of all cases, predominantly due to lung cancer [2]. Benign causes are non-malignant tumor compression, infectious disease or complications from indwelling intravascular devices [3]. The recently increased utilization of indwelling catheters and pacemaker/defibrillator leads have particularly led to a rise in device-related SVC syndrome [4, 5]. The exact numbers in Europe and worldwide are less well-known; however, the incidence of SVC syndrome reported in the literature ranges approximately from 1 in 650 to 1 in 3100 patients. Due to the lower number of hemodialysis catheters implanted, the incidence of catheter-related SVC obstruction may be lower in Europe, thereby shifting the ratio more toward a malignant etiology [6]. The most common causative tumor entity is bronchial carcinoma, followed by all tumors that frequently metastasize to the mediastinum as well as primary mediastinal tumors (such as thymomas or lymphomas). In contrast to SVC obstruction, IVC obstruction is infrequently referred to as IVC syndrome in the literature, since it is rarely associated with severe complications such as hypotension, tachycardia, end-organ failures and death [7]. The underlying pathologies leading to IVC obstruction are extremely diverse, which makes reliable estimates on epidemiology, especially toward the nature of these obstructions, virtually impossible. While 4–15% of IVC obstructions seem to be associated with acute deep venous thrombosis (DVT) and subsequent bilateral leg swelling [8], many IVC obstructions stay asymptomatic and are coincidentally diagnosed. Although different forms of malignant and benign pathologies are associated with IVC obstruction, these entities are mainly restricted to case reports and the exact incidence of IVC obstruction remains unclear [9]. The heterogenicity in reporting IVC obstructions causes further challenges to reliably determine the etiology and epidemiology. For example, a primary IVC thrombosis is uncommon and may be associated with a May–Thurner compression [10], Budd–Chiari syndrome [11] or as a proximal extension of an iliofemoral DVT [12]. An iatrogenic cause often in association with an implanted IVC filter is well-known [13, 14]*.* Congenital IVC obstructions have an estimated prevalence of 0.3–0.6% in the general population [8]. During embryogenesis, the IVC is formed by continuous formation and regression of the posterior cardinal, subcardinal and the supracardinal veins. Abnormal regression or persistence of any of these embryonic veins might cause IVC anomalies [15]. In the literature, these anomalies have inconsistently been referred to as agenesis, hypoplasia, aplasia or atresia. An in-depth analysis of the embryological development of the IVC is beyond the scope of this paper. In practice, however, the implications for endovascular therapy options are more important. Although the terminology may falsely suggest that endovascular treatment is not possible, a residual IVC structure can almost always be identified on magnetic resonance venography (MRV) or is identified during angiographic imaging and endovascular recanalization maneuvers, thus supporting recanalization efforts (e.g., Figure 1B).

## Clinical Presentation

### Superior Vena Cava Obstruction

The signs and symptoms depend on the temporal dynamic, degree and location of superior vena cava obstruction and range from slight discomfort in the upper extremities to a life-threatening complication of sustained venous congestion of the head and neck [4, 16].

When the development of an SVC obstruction progression is slow, collateral pathways may develop which facilitate adequate venous outflow from the upper extremities through compensatory pathways. This causes less profound symptoms such as swelling, discomfort, aching and heaviness or discoloration of the upper extremities. An acute problem exists when the speed of obstruction development exceeds the natural compensation of venous outflow. Mostly, this resembles an acute-on-chronic occlusion, i.e., thrombosis. Symptoms then worsen rapidly, and patients present with dyspnea, facial swelling, neck distension and cough developing over a relatively short period of time. In the most severe cases, cerebral, laryngeal and pharyngeal edema due to sudden elevation in venous pressures from rapidly occluding SVC may occur and demands immediate intervention. Yu B et al. proposed a classification system to assist in determining the urgency of intervention and tracking progression of symptoms [17] (see Table 1). The proposed management algorithm for superior vena cava syndrome, which categorizes treatment recommendations based on the severity of symptoms, emphasizes the importance of matching treatment intensity with symptom severity to optimize patient outcomes while avoiding unnecessary interventions.

**Table 1:** Proposed grading system for superior vena cava syndrome according to Yu B. et al. [17]

Grade	Category	Estimated Incidence (%)	Definition
0	Asymptomatic	10	Radiographic superior vena cava obstruction in the absence of symptoms
1	Mild	25	Edema in head or neck (vascular distention), cyanosis, plethora
2	Moderate	50	Edema in head or neck with functional impairment (mild dysphagia, cough, mild or moderate impairment of head, jaw or eyelid movements, visual disturbances caused by ocular edema)
3	Severe	10	Mild or moderate cerebral edema (headache, dizziness) or mild/moderate laryngeal edema or diminished cardiac reserve (syncope after bending)
4	Life-threatening	5	Significant cerebral edema (confusion, obtundation) or significant laryngeal edema (stridor) or significant hemodynamic compromise (syncope without precipitating factors, hypotension, renal insufficiency)
5	Fatal	< 1	Death

A different scoring system for signs and symptoms of SVC syndrome was established in 1993 by Kishi K et al. in order to assess treatment response after stenting [18].

### Inferior Vena Cava Obstruction

Although IVC syndrome as a result of IVC obstruction has been described [19], it is less frequently found than SVC syndrome. In general, IVC obstruction rarely evolves into a life-threatening condition. Nevertheless, it may still give rise to significant physical restrictions with severe reduction in quality-of-life (QoL) [20–22]. Phlegmasia cerulea dolens is a rare but potentially fatal complication of iliofemoral thrombosis that may originate from an acute IVC obstruction, especially if the patient has an indwelling IVC filter [23]. A critical clinical presentation is uncommon in IVC narrowing which gradually develops, due to the broad potential for building collateral pathways, either through the azygos/hemiazygos system, abdominal wall collaterals or systemic venous shunts[24]. In cases of congenital IVC obstruction, the obvious clinical signs and symptoms occur when a thrombosis of IVC remnants or venous collaterals develop [8].

Bilateral lower extremity symptoms may be more indicative of a chronic IVC obstruction without thrombosis. Leg swelling, venous claudication and in chronic or severe cases, discolorations and ulcerations could direct toward a (chronic) IVC obstruction. In addition to these sometimes difficult to interpret symptoms, prominent superficial venous structures visual in the abdominal wall, groin or pubic area have been suggested to be pathognomonic for a significant IVC obstruction [26]. A definite diagnosis, however, can only be established with adequate imaging techniques.

## Pre-procedure Imaging

Despite being operator-dependent [27], ultrasound is often preferred for detecting acute thrombosis in jugular, subclavian, femoral and popliteal veins [28, 29]. However, its effectiveness in identifying iliocaval obstruction is debated [30, 31]*.*

Conventional venography, though still useful in certain cases and for guiding endovascular interventions, has been largely replaced by cross-sectional imaging techniques such as CT and MRI due to their superior sensitivity and specificity [32]. CT of the chest, with a diagnostic sensitivity of 96% and specificity of 92%, is preferred for the SVC over MRI, which can be affected by pacemaker leads and mediastinal movements [33]. Conversely, MRI is advantageous for IVC imaging due to the absence of ionizing radiation, making it suitable for young patients. MR venography offers high-resolution imaging and can accurately estimate thrombus age, aiding in the decision for endovascular thrombectomy [34].

**Fig. 2:**
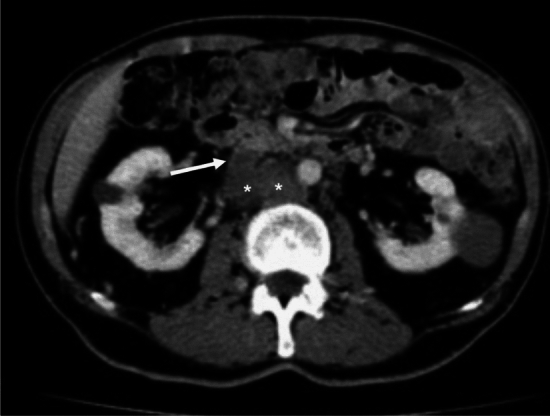
Axial CT image. Example of an acute thrombosis of a chronically obstructed IVC (arrow) with surrounding thrombosed venous lakes (asterisks), falsely diagnosed as a retroperitoneal malignancy

Additionally, MR venography can detect small IVC remnants overlooked by CT and differentiate between various etiologies, such as distinguishing dilated venous collaterals from retroperitoneal malignancies (See Figs. 1A, B and 2). Ultimately, the choice of diagnostic imaging depends on personal experience and institutional circumstances, and the methods should be used complementarily for a comprehensive assessment. Details are addressed elsewhere.

## Endovascular Therapy

For many years, endovascular treatment was not the preferred strategy to treat SVC and IVC obstructions. This was mainly due to the limited availability of interventional devices. With the development of new techniques that facilitate recanalization and devices supporting long-term patency, endovascular treatment is currently considered the mainstay strategy for chronic vena cava obstructions.

### Superior Vena Cava

Venous access for endovascular management of SVC obstruction should be as close to the lesion as possible to improve pushability and technical success. The internal jugular vein (IJV) is typically preferred, though additional access sites such as the cephalic or basilic veins may be necessary for Y-stenting configurations [35, 36] (see Fig. 2). A unilateral or bilateral therapy depends on several factors such as location of the underlying pathology and the clinical presentation (e.g., swelling of one or both arms).

A combination of femoral and jugular or upper extremity venous access is often the most secure, facilitating techniques such as "body floss” [37]. Stent placement can also be more comfortably performed from the common femoral vein access.

Despite high technical success rates from both retrograde and antegrade approaches, recanalization can be challenging. Stiff hydrophilic guidewires with curved support catheters, long sheaths and triaxial systems can help. Multiple C-arm angulations during wire advancement and venography are needed to confirm guidewire positioning. Simultaneous balloon venoplasty of two parallel balloons and snaring techniques can connect different lumens. If conventional techniques fail, aggressive methods such as sharp recanalization [38] or radiofrequency wires may be used, although these are associated with increased risks [39, 40].

**Fig. 3:**
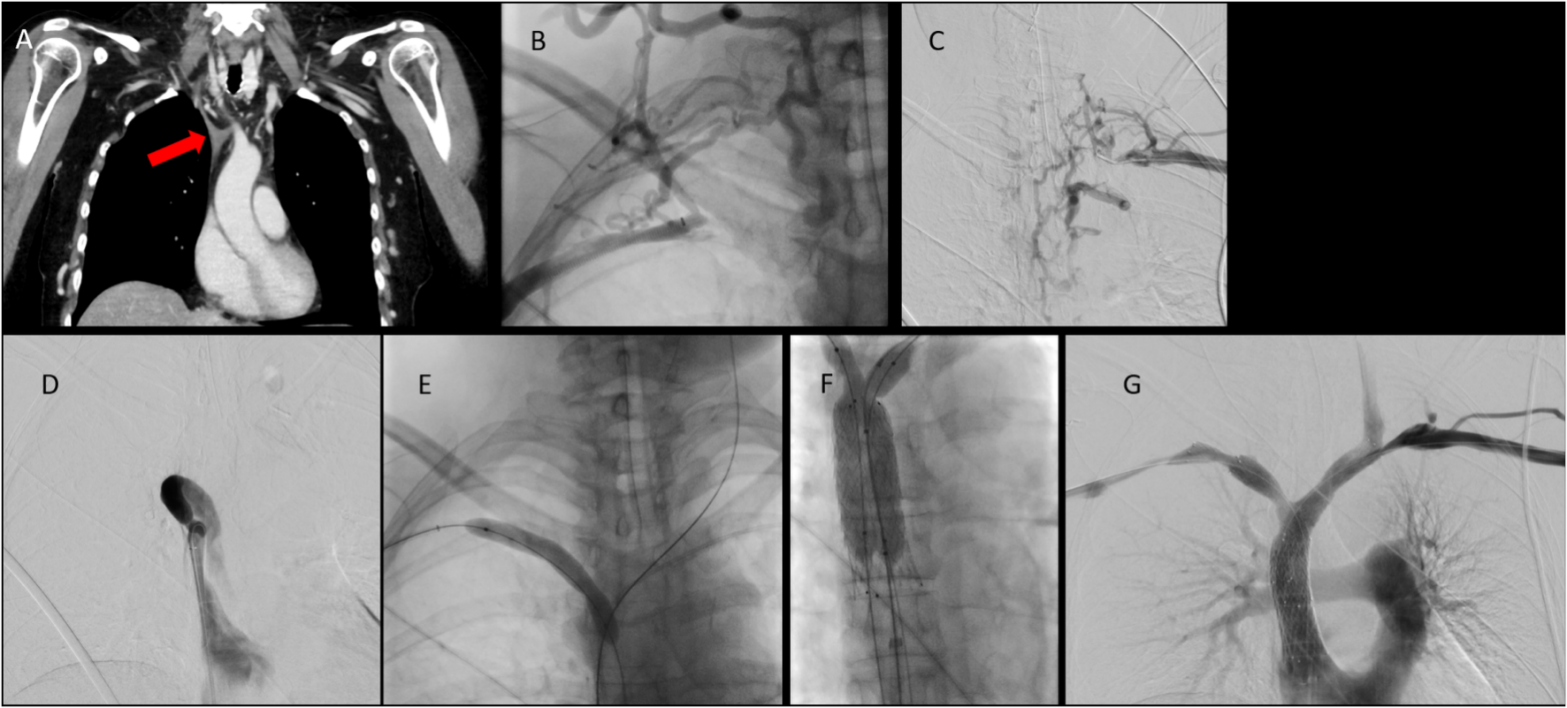
43-year-old female patient with short bowel syndrome and multiple port-a-cath implantations over the last years presenting with chronic occlusion of the brachiocephalic vein on both sides as well as the superior vena cava, resulting in a severe post-thrombotic superior vena cava syndrome; **A**: Coronal CT image showing the occlusion of the SVC and both brachiocephalic veins (red arrow in A). **B** and **C**: Right and left brachial venous access with phlebography confirming the occlusions from the right (B) and left side (C). **D**: Occlusion of the SVC from the caudal (femoral) access. **E**: Wire passage from anterograde and retrograde. Two flossing wires in place. Ballooning of the right brachiocephalic vein. **F**: Y-shaped reconstruction the occlusion of SVC with one 20 mm Venovo Stent (BD Bard) and two 12 mm Venovo Stents in each brachiocephalic vein. **G**: Final phlebography showing the SVC reconstruction

After establishing a stable guidewire position, vessel preparation is performed. Although balloon-expandable stents were used initially, bare metal self-expanding stents are now preferred and thorough predilation is recommended [41]. Caution is advised due to the risk of vein rupture in malignancy-related obstruction (Fig. 3), especially after radiation therapy [42, 43].

There is no scientifically based recommendation on a superior stent design or configuration. Initial experiences primarily involved Wallstent and Z-stent [44], with newer venous stents now available but mostly used in iliac veins [45, 46]. Adequate sizing is crucial, particularly in malignancy cases, to prevent stent migration after radiotherapy or chemotherapy. Covered stents may be effective but are limited by potential obstruction of important collaterals [41].

Stent placement is typically performed under conscious sedation and local anesthesia but may require general anesthesia for lengthy procedures or patient discomfort [47](Fig. 2). Anticoagulation therapy is common during and after the procedure, though the benefits for stent patency are not well established [48]. Different anticoagulants have been used inconsistently, with no proven advantage in stent patency [49, 50].

### Inferior Vena Cava

The interventional techniques required for IVC recanalization do not differ much from those described above for SVC recanalization. Obviously, the primary access site changes to the (common) femoral vein, since the distance to the IVC is shorter. Furthermore, traveling across the right atrium coming from an IJV approach might decrease pushability. Nevertheless, the additional IJV access may again help to achieve a through-and-through wire to enable the “body floss” technique, which may be helpful for passing catheters and balloon through the long and severely scarred cavo-iliofemoral segments. Alternative access sites include the popliteal vein; however, this may only be beneficial when thrombectomy for acute iliofemoral DVT is considered. Mechanical thrombectomy or catheter-directed thrombolysis (CDT) should precede venoplasty and stenting in cases of acute thrombotic appositions (see Fig. 1C).

**Fig. 4:**
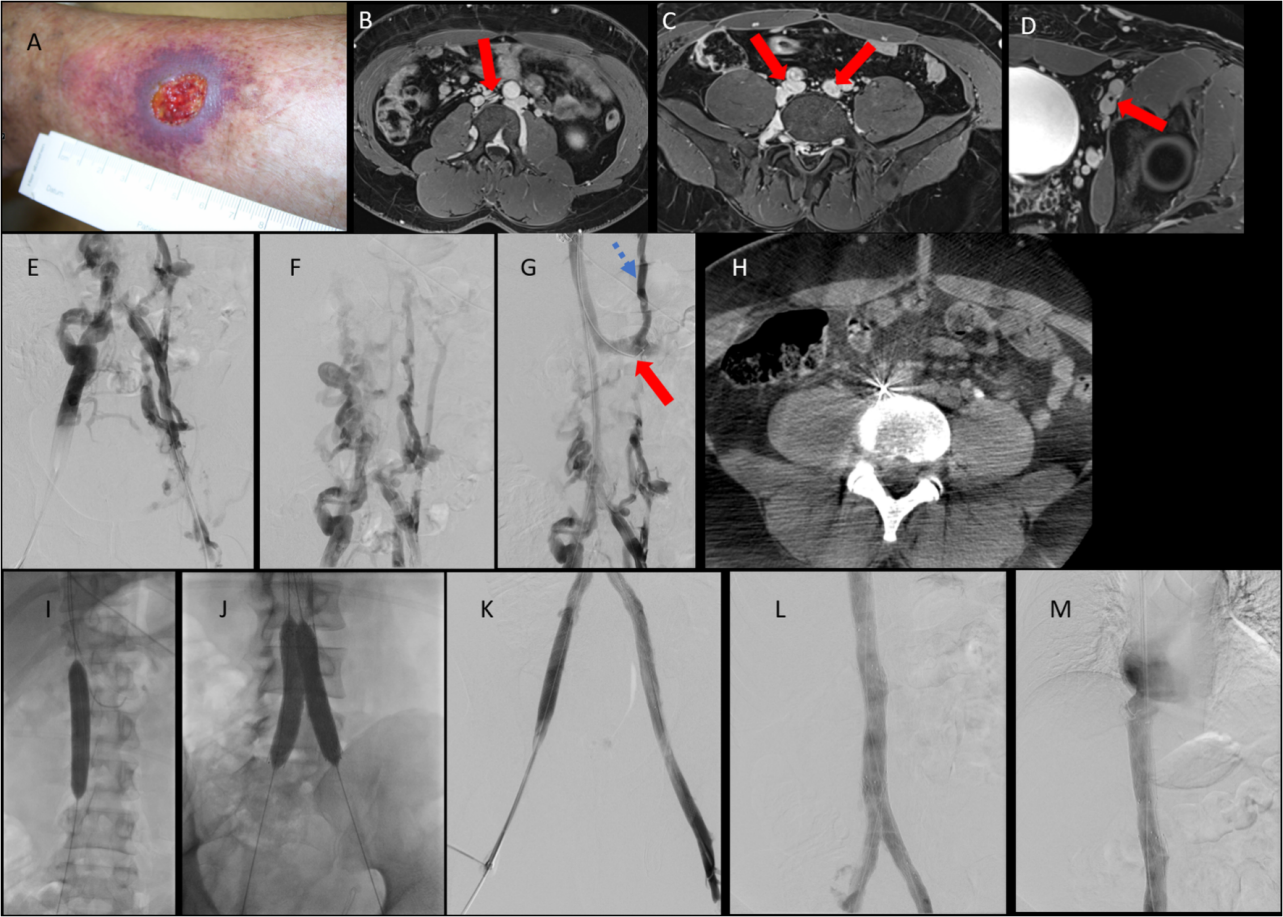
40-year-old male patient with severe post-thrombotic syndrome and ligation of the inferior vena cava. **A**: Clinical image of the left leg, with swelling and venous ulcer. **B**: MR venography with IVC remnant (red arrow) and venous lakes **C** and **D**: iliac veins with post-thrombotic synechiae down to the level of the left external iliac vein. **E** and **F**: Corresponding venography images of the diseased caval confluence (E) and lumbar collaterals (F). G: Wire passage of the occlusion after access via right and left femoral vein and right IJV. Catheter in the left renal vein (red arrow) with partial venous drainage of the left kidney via collaterals (blue dotted arrow). **H**: Cone-beam CT confirming wire path in the IVC remnant. **I**: ballooning of the occlusion to prepare stent implantation. **J**: Reconstruction of the IVC confluence using two parallel 14 mm self-expanding Venovo Stents (BD Bard) in a large 24mm Sinus XL Stent. Hypoplastic retrohepatic IVC was stented using a 28 mm Sinus XL Stent (not shown) to ensure outflow. **K, L** and **M**: Final venogram after reconstruction of the iliac vein (K), iliocaval confluence and distal IVC (L) and retrohepatic IVC (M)

Similar to SVC recanalization, thorough balloon venoplasty as a vessel preparation for subsequent stent placement is recommended (See Fig. 4I). Significant bleeding after venoplasty of a chronically obstructed IVC rarely occurs and is less likely to cause life-threatening hemorrhage [51].

Conventional balloon venoplasty generally suffices to prepare chronic IVC lesions before stenting. There is no supporting evidence for the application of cutting balloons, scoring balloons and drug-eluting balloons in the IVC. In the very unlikely case that a lesion cannot be dilated with conventional balloons, a high-pressure balloon may be considered.

After venoplasty, the use of intravascular ultrasound (IVUS) may be considered. The additional value of IVUS in the diagnosis and treatment of deep venous disease has been repeatedly reported [52, 53].

For IVC reconstruction, IVUS may have a role in determining stent position. A proportional stent size may be derived from adjacent venous segments, but it should be recognized that lack of flow or local dilatation due to venous hypertension may give false measurements.

**Fig. 5:**
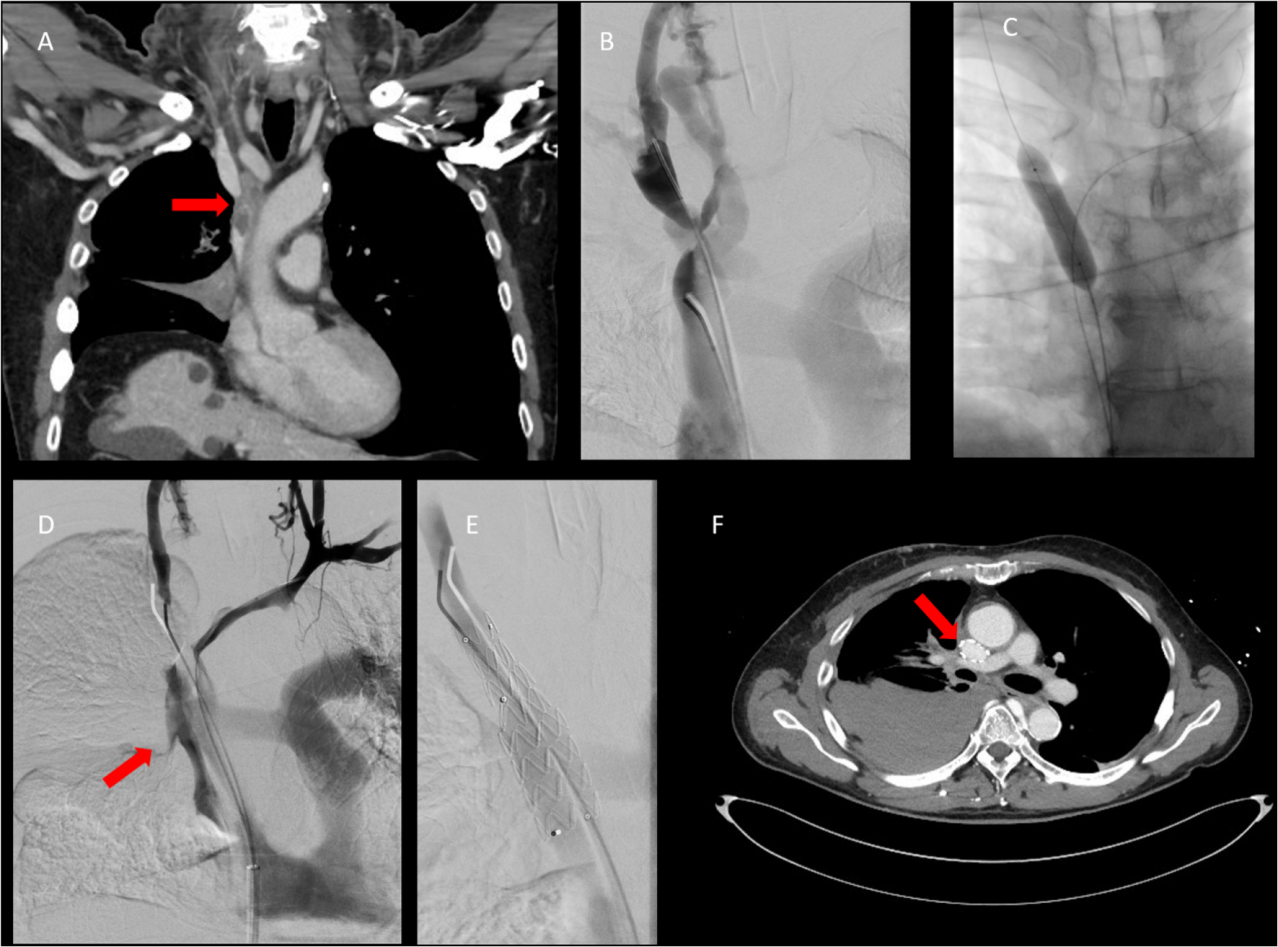
70-year-old male patient with bronchial adenocarcinoma and tumor-related high-grade stenosis of the superior vena cava with signs of SVC syndrome. **A**: Coronal contrast-enhanced CT showing near complete occlusion of the superior vena cava; **B**: Phlebography showing extensive venous bypass circulation; **C**: Ballooning of stenosis with a 12 mm balloon; **D**: Iatrogenic rupture of the superior vena cava (red arrow) with bleeding in the pleural cavity; **E**: Immediate implantation of a 20 mm endograft (Medtronic Endurant) at the rupture site with subsequent control; **F**: Axial slice of the control-CT; right-sided hemothorax with well-deployed endoprosthesis in the superior vena cava (red arrow)

In cases of a (partly) thrombotic occlusion, IVUS may be used to determine the result after thrombectomy and guide further maneuvers until all segments are free from thrombus. Thereafter, the underlying chronic lesion can be identified and treated accordingly with stenting (Fig. 5).

Principally, bare metal self-expanding nitinol stents are nowadays used to recanalize the IVC. Although the first experience involved the Wallstent and some efforts were made to use arterial nitinol stents, the aggregated ideal characteristics of dedicated venous stents propelled these devices toward general acceptance for deep venous stenting procedures. In short, the desired qualifications should incorporate high radial force/crush resistance, sufficient flexibility and large diameters. These combined characteristics cannot be found in arterial designed stents, although what exactly exemplifies ideal radial force or flexibility is not known. The recommended diameter of stents implanted in the IVC is 18- 24 mm [54]*.*

Although infrequent, stent migration is a feared complication and may be avoided by a 2 mm oversizing of stents. After stenting, IVUS may again be valuable to evaluate for residual stenosis, correct stent position and early re-thrombosis.

The inflow of the renal veins may be in the diseased segment of the ICV. Although a large body of evidence about the relevance of jailing the renal inflow is lacking, available data suggest that renal outflow and function may be preserved even using self-expanding nitinol stents [55].

In contrast to the SVC, short stenoses are less likely in the IVC and the procedure time and long segment venoplasty up to large diameters may be uncomfortable. Therefore, general anesthesia may be helpful in most if not all recanalization procedures.

Although intra-procedure anticoagulation is usually with intravenous heparin, the antithrombotic regimen after IVC stenting has not found consensus. In a recent systematic review on IVC stenting, it was found that post-procedure anticoagulation varied widely from no anticoagulation to full anticoagulation with warfarin and dual antiplatelet therapy [51].

Similar challenges arise when aiming to extrapolate an antithrombotic regimen from chronic deep venous obstruction therapy in general. In the recently published ESVS clinical practice guidelines, the peri- and post-interventional antithrombotic regimens were found to be heterogeneous among studies. No particular strategy on either duration or type of anticoagulation could be recommended [56]. In a Delphi consensus paper, anticoagulant treatment should be continued for at least six months after intervention in patients with a history of DVT. Regarding the role of antiplatelets, no consensus was reached [57].

## Post-interventional considerations

Thrombosis is the most common cause of stent patency loss. Although this process is multifactorial, inadequate antithrombotic regimen or non-compliance is likely to be an important provocation. Another significant contributor to stent re-occlusion is suboptimal inflow into the stents or an outflow obstruction, usually by preexistent disease not completely covered by the implanted stent [58]. With the introduction of dedicated venous stents, foreshortening and other stent-related problems have been reduced and ample use of IVUS may prevent overlooking significant obstructions [59].

Clear follow-up recommendations for SVC and IVC interventions are lacking. Again, deriving recommendations from experience with chronic deep venous obstructions, a baseline duplex ultrasound is required on the first day after the procedure. Because a thrombosed stent can be recanalized with thrombolysis within 14 days of the thrombotic event, and most thrombotic complications occur shortly after the intervention, a second ultrasound is recommended within two weeks after stent placement. Further follow-up visits are scheduled at six weeks, three and six months and yearly [56]. Routine cross-sectional imaging is not recommended. Indications for re-intervention include acute in-stent thrombosis or symptomatic in-stent restenosis > 50%. Options for re-intervention are not different from the endovascular therapy for acute DVT. The application of available pharmaco-mechanical thrombolysis techniques should be dictated by personal experience. Additional re-intervention techniques include venoplasty and re-stenting [56]. Concerning SVC obstructions, no specific follow-up is required, since a restenosis/occlusion will cause recurrent symptoms.

## Results

For both superior vena cava and inferior vena cava obstructions, the existing literature is constrained by a lack of studies with large patient cohorts, prospective designs and standardized treatment and reporting protocols.

### Superior Vena Cava

A recent systematic review and meta-analysis have been conducted to evaluate the endovascular treatment of SVC obstructions, contributing valuable insights to this area of research [2]. While the systematic review included an acceptable number of studies, the heterogeneity of the data significantly complicated the ability to draw definitive conclusions. The studies varied widely in terms of the number of subjects, the indications for treatment and the types of stents used—most of which were not specifically designed for venous application. Nevertheless, it is evident that the technical success rate of endovascular recanalization of the SVC is very high, approaching nearly 100%.

These findings were re-affirmed by a recent meta-analysis including 39 studies evaluating the efficacy and safety of endovascular therapy for superior vena cava syndrome; a high technical success rate of endovascular therapy (98.8%) and low rates of restenosis (10.5%) and recurrence (10.8%) were recorded [48]. Complications in general were minor and reported with a rate of 8.6%. However, prognosis of SVC syndrome is strongly influenced by the underlying disease.

An older meta-analysis from 2006 and a 2014 review identified that percutaneous stenting of the superior vena cava in cases of superior vena cava syndrome exhibits primary patency rates ranging from 64 to 95 percent. Additionally, these studies reported secondary patency rates of 93 to 100 percent, recurrence rates between 0 and 40 percent, complication rates from 0 to 25 percent and mortality rates of approximately 3 to 4 percent [60, 61].

In terms of clinical outcomes for superior vena cava, stent placement is quite effective for symptom relief, with up to 97–99% of patients experiencing rapid postoperative relief [62, 63].

Short-term success rates were also generally favorable, reported up to 92%, although the range was broad, spanning from 53 to 100%. The absence of standardized treatment and reporting protocols was further underscored by the moderate to high heterogeneity observed in the summarization of primary and secondary patency rates across the studies [64]. Despite the noted heterogeneity in the data, the endovascular approach remains the preferred treatment for symptomatic superior vena cava obstructions, supported by a weighted mean patency rate of 95% and relatively low major complication rates of approximately 4%. These findings are corroborated by another recent review, which reported a pooled technical success rate of 96.8% (95% CI 96.0–97.5%) and a clinical success rate of 92.8% (95% CI 91.7–93.8%), reinforcing the efficacy of this treatment modality [65]. While specifically examining benign superior vena cava syndrome, both technical and clinical success rates were uniformly reported at 88.8% (95% CI 83.0–93.1%) [65].

Additionally, pooled data indicated that patency rates remained above 90% during the first year following treatment.

Data about the choice of stents are sparse. However, a recent prospective study on malignant occlusion found that covered stents retained patency longer than non-covered stents, with a 12-month patency rate of 94 versus 48% for covered versus non-covered, respectively [63].

### Inferior Vena Cava

Publications focusing on inferior vena cava obstruction are notably rarer and exhibit the same limitations as previously described for SVC obstruction studies. Furthermore, the inclusion of cases involving (bilateral) thrombosis and confluence stenting complicates the analysis, diluting the data pertaining specifically to straightforward IVC stenting [64, 66]. Interestingly, patency data for IVC recanalization are comparable to that of the SVC when the obstruction is related to malignancy (ranging from 68% to 96.4%). However, when the obstruction is benign in origin, both primary and secondary patency rates for IVC obstructions are notably lower (ranging from 57 to 91%) [67]. The lower patency rates observed in benign IVC obstructions could be attributed to factors such as the extended length of the obstruction, the number of stents required or the configuration of confluence stenting that is often necessary when the iliac veins are implicated. Additionally, patients with benign conditions frequently exhibit post-thrombotic lesions in the iliac veins, which necessitate the use of long stents to ensure comprehensive treatment. Importantly, there is no evidence to suggest that crossing a vena cava filter during IVC stenting adversely affects patency rates.

In conclusion, endovascular recanalization of the SVC and IVC is a well-established technique characterized by high technical and clinical success rates, along with an acceptable risk profile. The ongoing advancements in deep venous interventions are likely to continue enhancing the outcomes of SVC and IVC procedures. However, there is a notable lack of evidence regarding the optimal post-interventional antithrombotic regimen, highlighting a critical area for future research.
