# Correction to: Inferior and Superior Vena Cava Reconstruction

**DOI:** 10.1007/s00270-025-03989-w

**Published:** 2025-03-11

**Authors:** Rick de Graaf, Arne Estler, Gerd Grözinger

**Affiliations:** 1 Department of Diagnostic and Interventional Radiology, Clinic of Friedrichshafen, Friedrichshafen, Germany; 2 Department of Diagnostic and Interventional Neuroradiology, University Hospital Tübingen, Hoppe-Seyler-Str. 3, 72076 Tübingen, Germany; 3 Department for Diagnostic and Interventional Radiology, University Hospital Tübingen, Tübingen, Germany

**Correction to: Cardiovasc Intervent Radiol (2024) 47:1616–1625** 10.1007/s00270-024-03867-x

The article “Inferior and Superior Vena Cava Reconstruction”, written by Arne Estler, et al., was originally published under exclusive license to Springer Science + Business Media, LLC, part of Springer Nature and the Cardiovascular and Interventional Radiological Society of Europe (CIRSE). As a result of the subsequent decision to publish the article under the open access model, the article’s copyright notice was changed on 28 February 2025 to © The Author(s) 2024 and the article is now distributed under a Creative Commons Attribution 4.0 International License, which permits use, sharing, adaptation, distribution and reproduction in any medium or format, as long as you give appropriate credit to the original author(s) and the source, provide a link to the Creative Commons licence, and indicate if changes were made.

The images or other third party material in this article are included in the article’s Creative Commons licence, unless indicated otherwise in a credit line to the material. If material is not included in the article’s Creative Commons licence and your intended use is not permitted by statutory regulation or exceeds the permitted use, you will need to obtain permission directly from the copyright holder. To view a copy of this licence, visit http://creativecommons.org/licenses/by/4.0.

